# Supplementary material for: Identification of a RAB32-LRMDA-Commander membrane trafficking complex reveals the molecular mechanism of human oculocutaneous albinism type 7
Source: bioRxiv. 2025 Feb 4:2025.02.04.636395. Preprint. [Version 1] doi: 10.1101/2025.02.04.636395 (PMC11838575; doi:10.1101/2025.02.04.636395)

Raw blots for Figure 1

1D

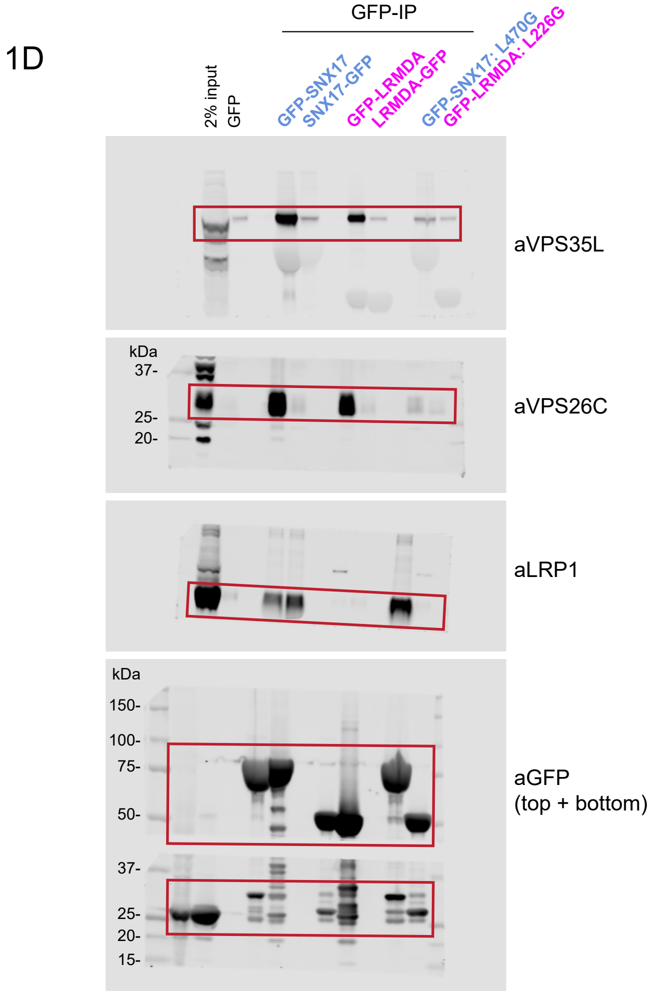

Raw blots for Figure 2

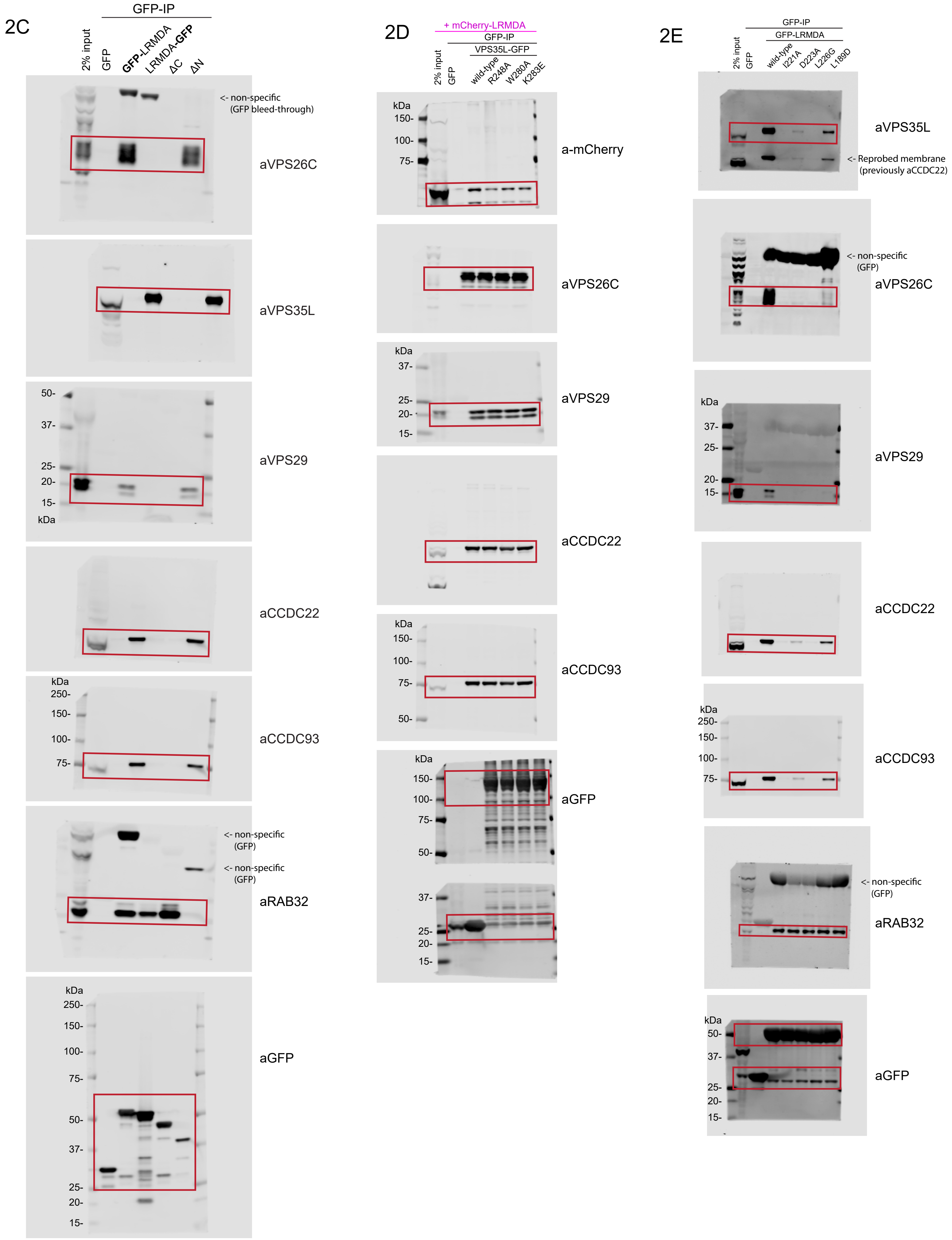

Raw blots for Figure 3

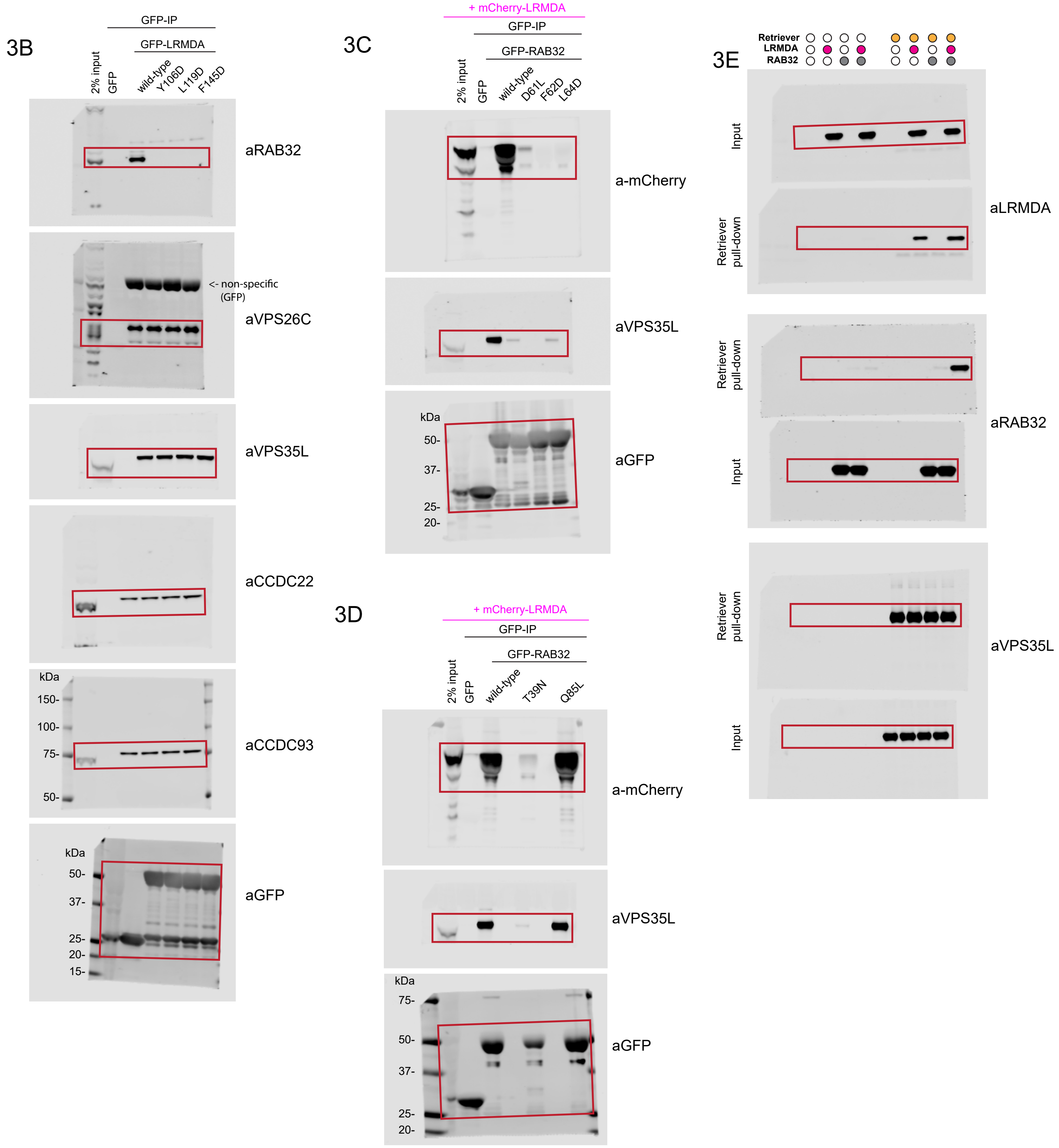

Raw blots for Figure 4

4E

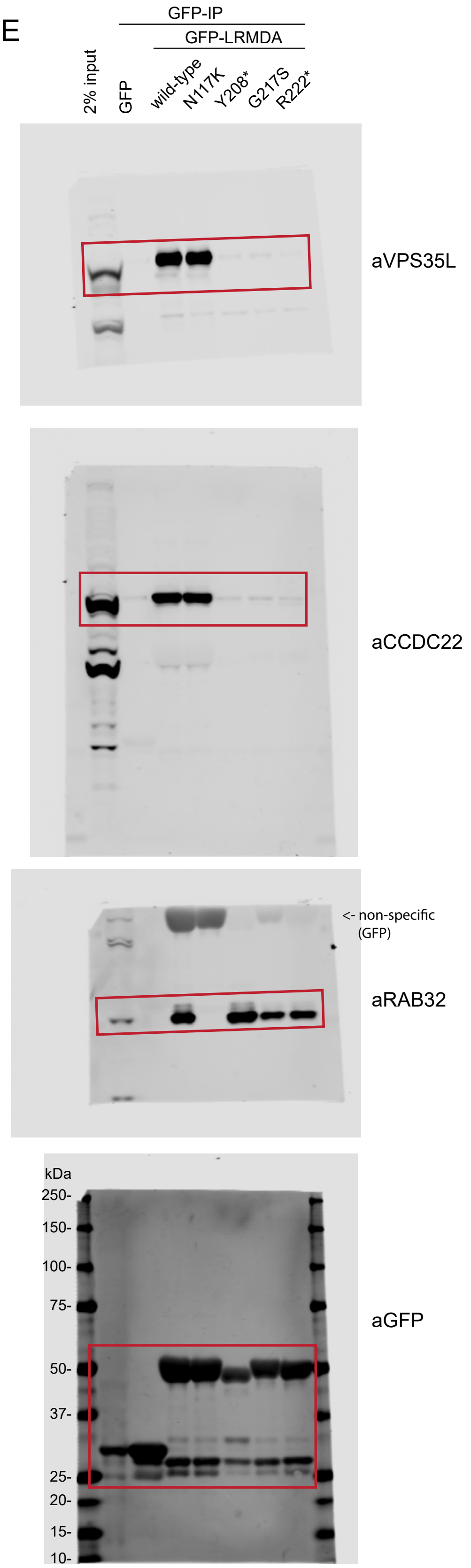

4F

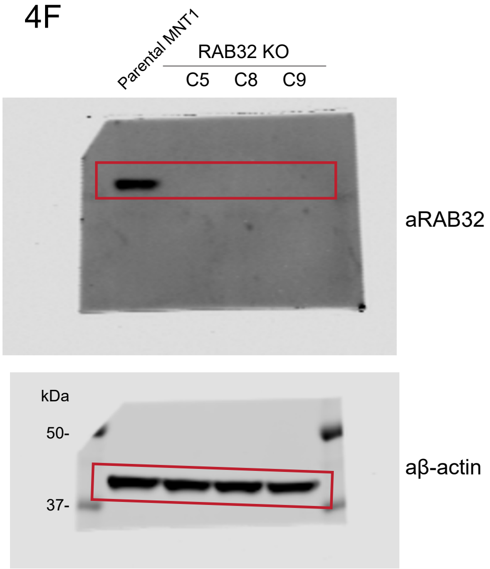

4I

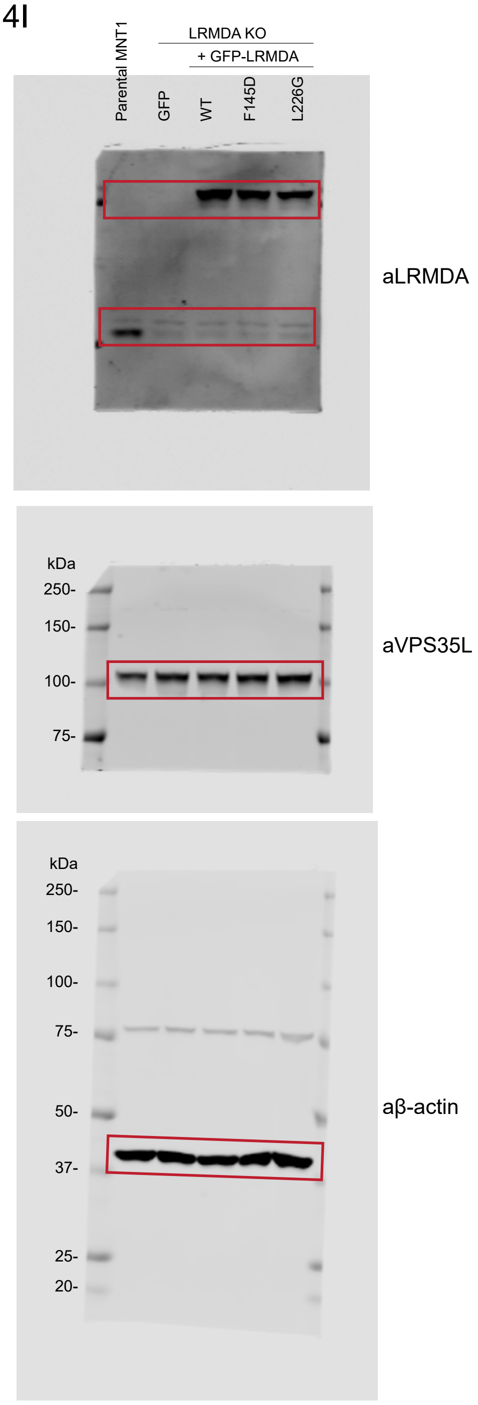

Raw blots for Figure 5

5A

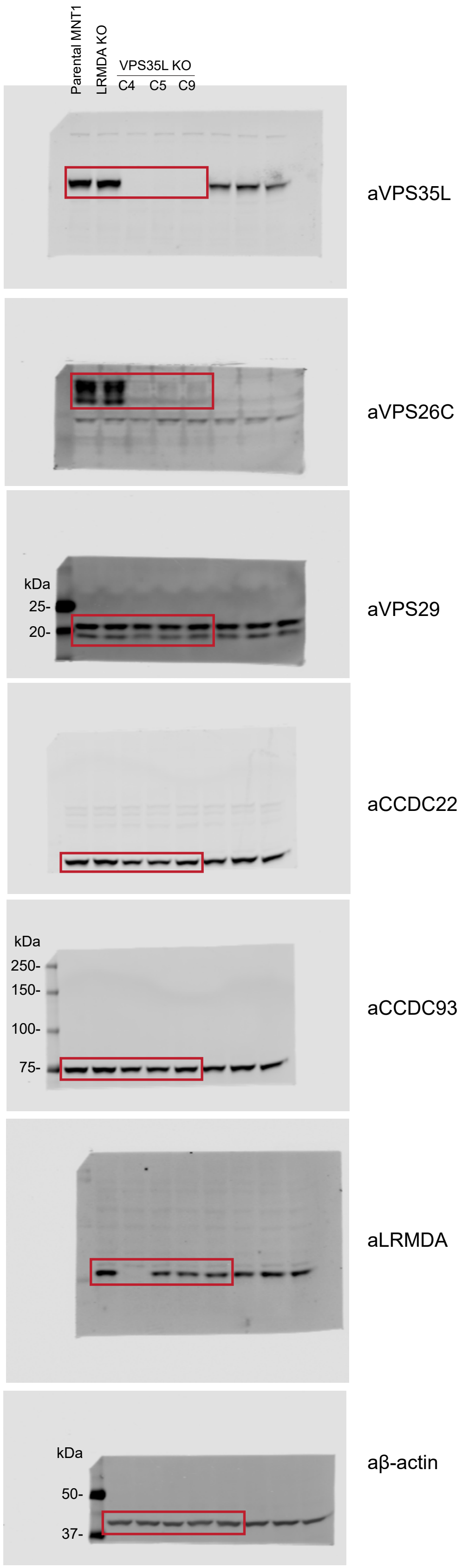

Raw blots for Figure 6

6B

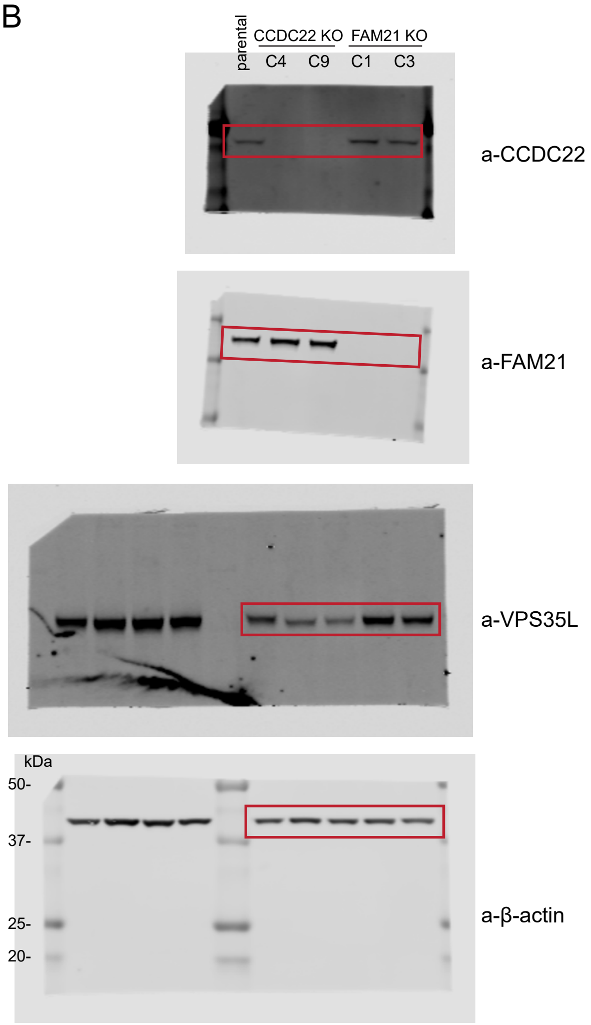

6E

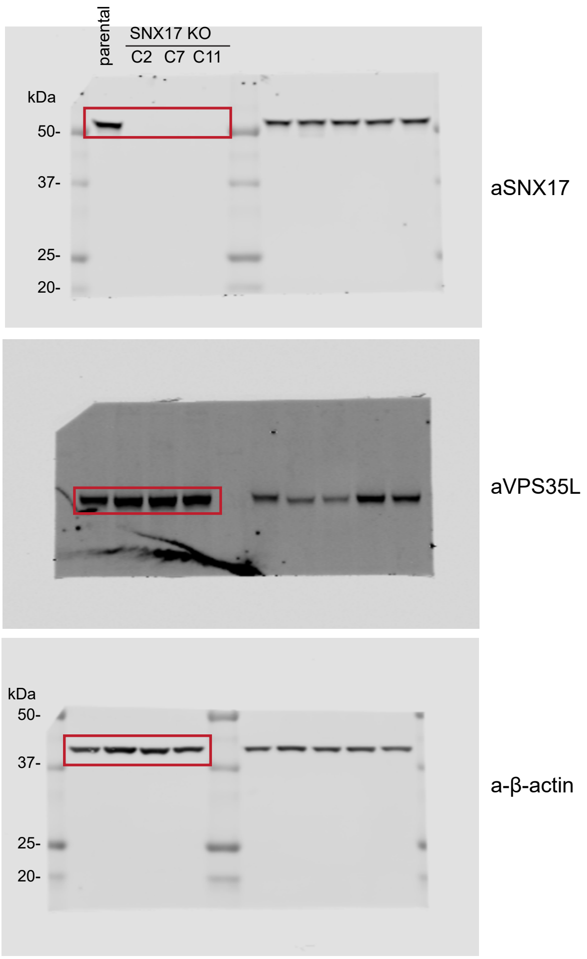

Raw blots for Figure 7

7B

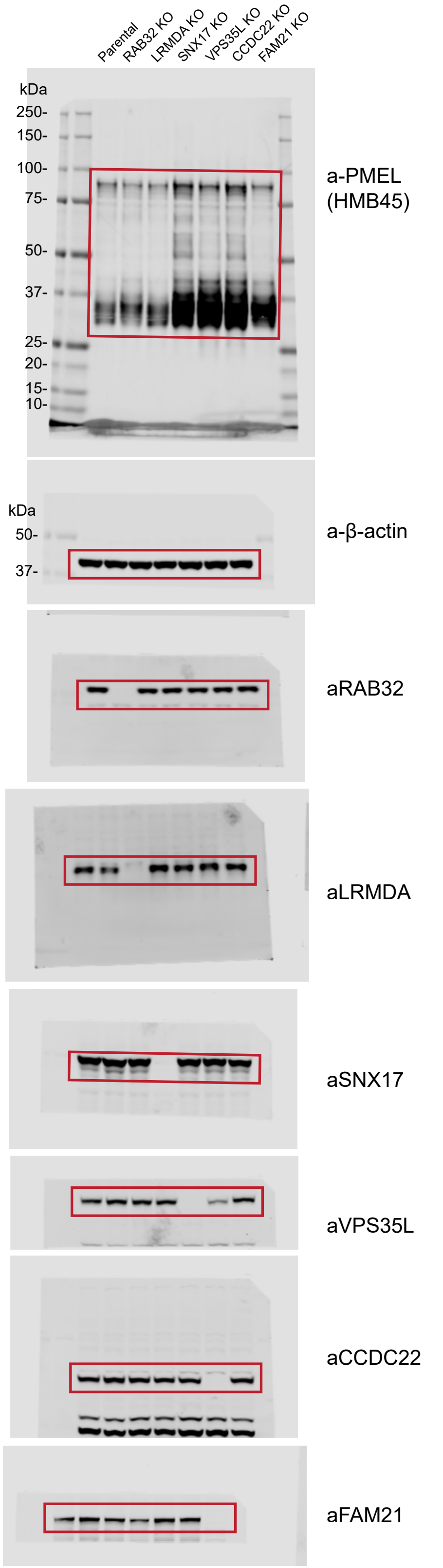

Raw blots for Supplementary Figure 2

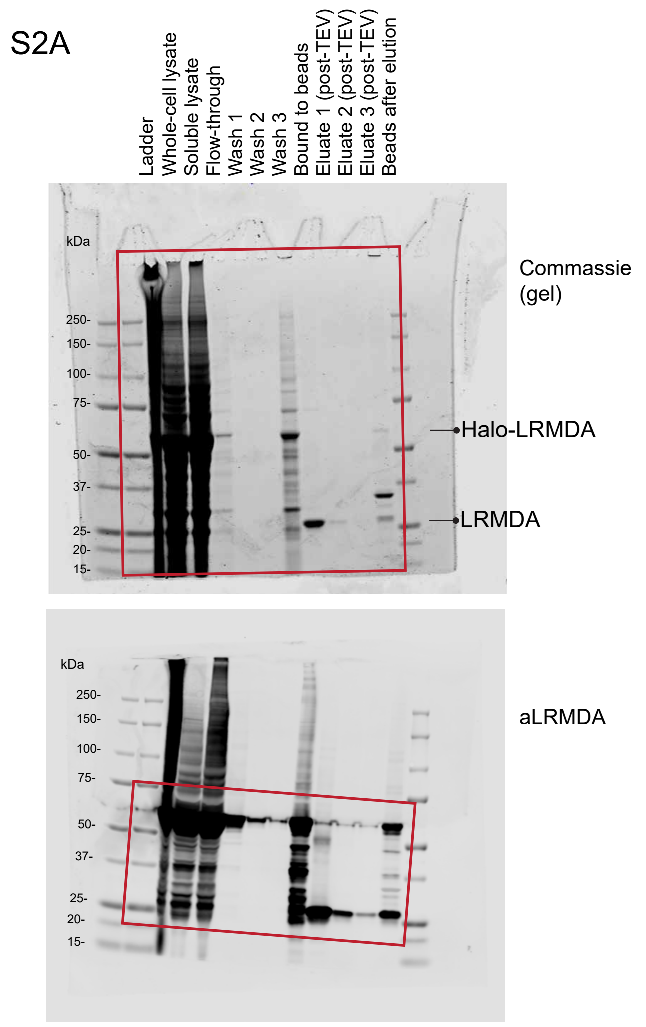

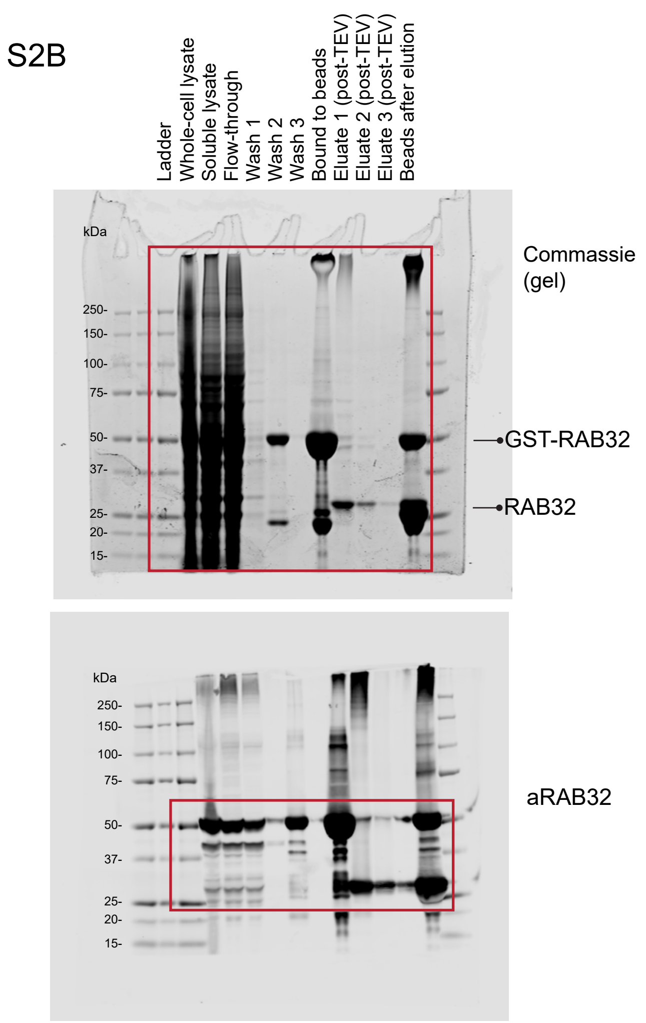

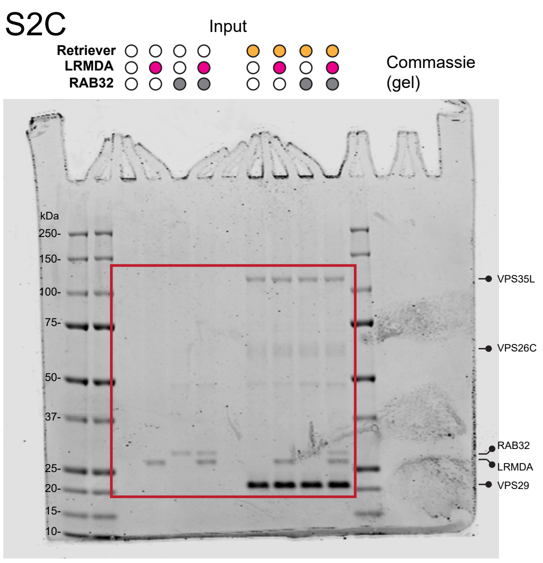

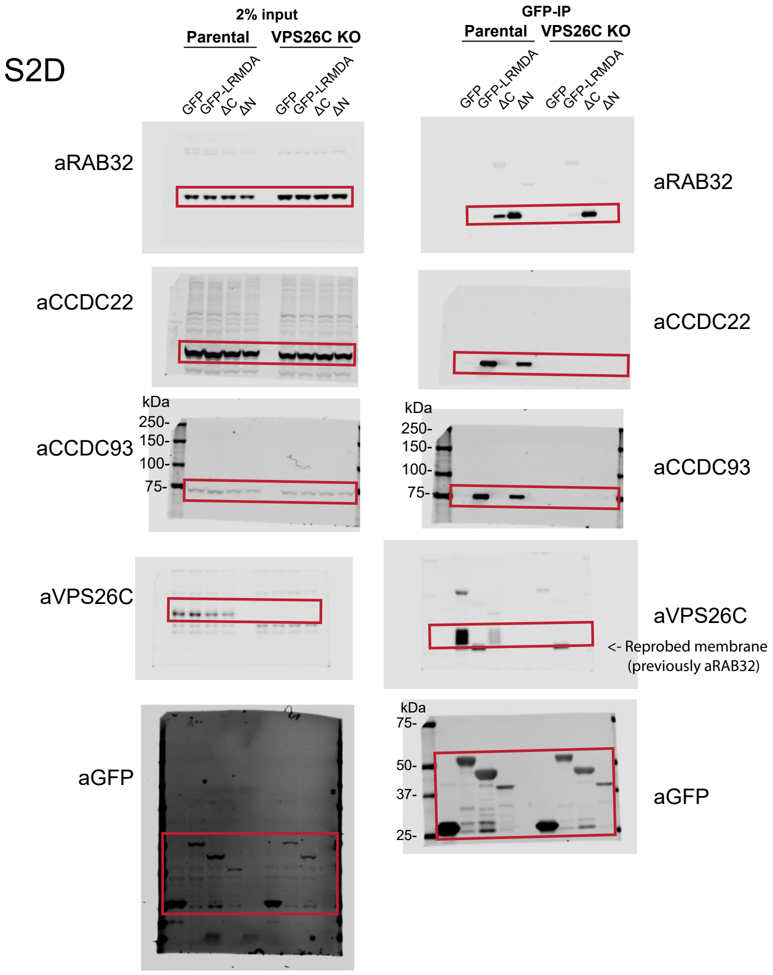

Raw blots for Supplementary Figure 3

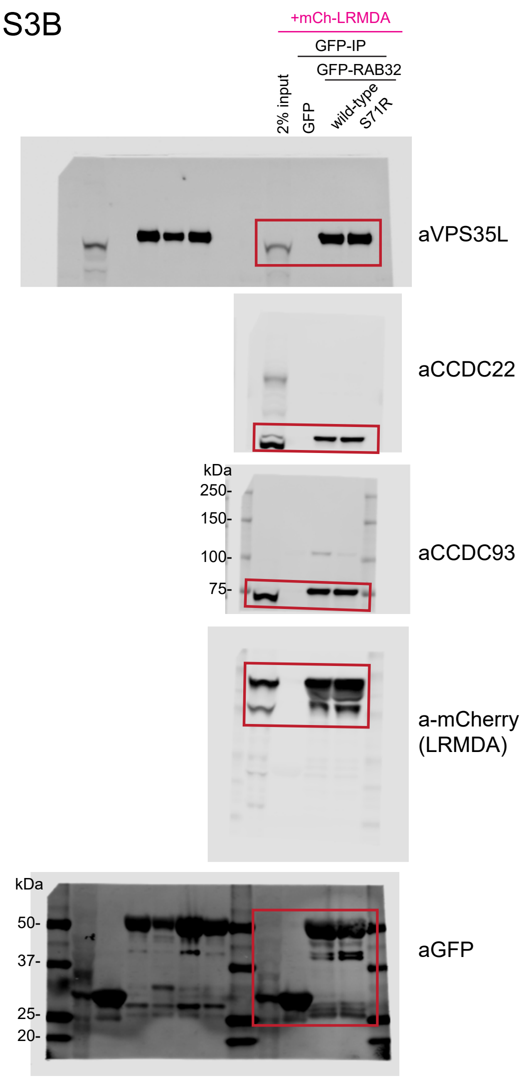

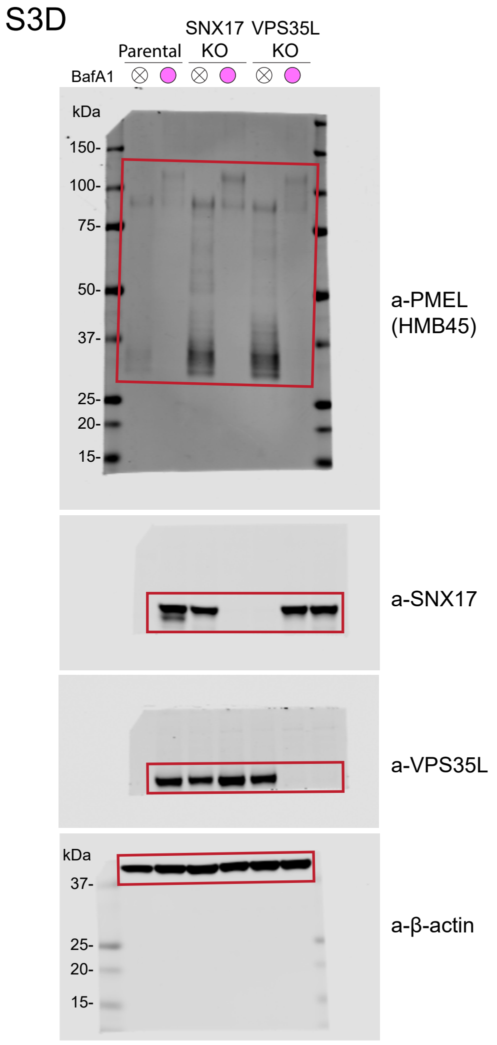

Supplement: Supplement 1 [file media-1.pdf]
