# Supplementary material for: Identification of a RAB32-LRMDA-Commander membrane trafficking complex reveals the molecular mechanism of human oculocutaneous albinism type 7
Source: bioRxiv. 2025 Feb 4:2025.02.04.636395. Preprint. [Version 1] doi: 10.1101/2025.02.04.636395 (PMC11838575; doi:10.1101/2025.02.04.636395)
Supplement: Supplement 3 [file NIHPP2025.02.04.636395v1-supplement-3.pdf]

bioRxiv preprint doi: https://doi.org/10.1101/2025.02.04.636395; this version posted February 4, 2025. The copyright holder for this preprint (which was not certified by peer review) is the author/funder, who has granted bioRxiv a license to display the preprint in perpetuity. It is made available under aCC-BY 4.0 International license.

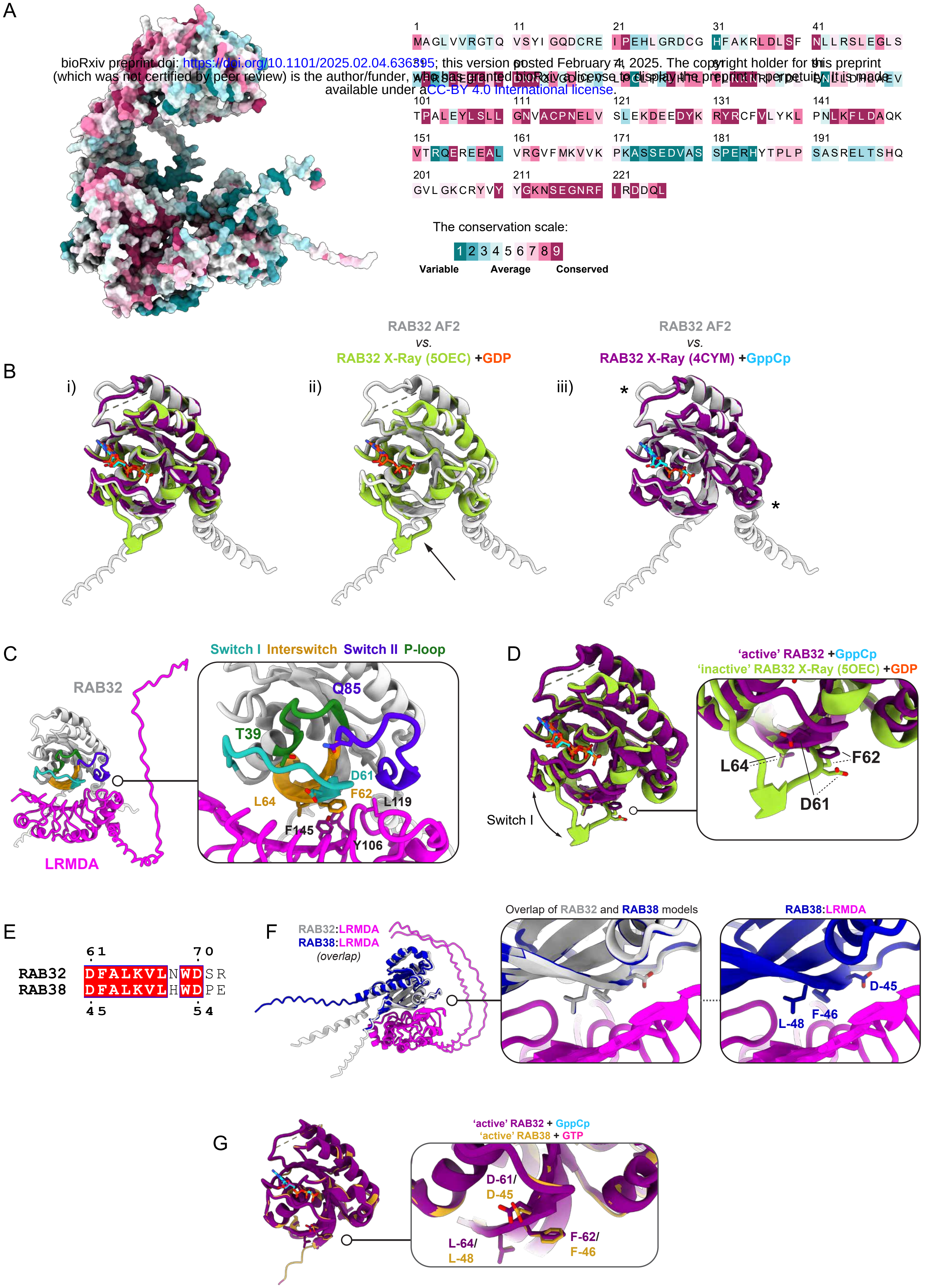

bioRxiv preprint doi: https://doi.org/10.1101/2025.02.04.636395; this version posted February 4, 2025. The copyright holder for this preprint (which was not certified by peer review) is the author/funder, who has granted bioRxiv a license to display the preprint in perpetuity. It is made available under aCC-BY 4.0 International license.

Supplementary Figure 1:

A) Evolutionary conservation of RAB32-LRMDA-Retriever assembly (Left) and LRMDA (Right) as determined by ConSurf tool.

B) Comparsion of AlphaFold2-predicted RAB32 conformation with experimentally resolved structures of (ii) inactive GDP-bound (PDB: 5OEC) (Wachtel et al., 2018), or (iii) active GppCp-bound RAB32 (GppCp is a non-hydrolisable GTP analog; PDB: 4CYM) (Hesketh et al., 2014). The depiction shows beter alignment with the active RAB32, and minor conformational differences are marked with stars. The conformation difference is more apparent in comparison to GDP-bound RAB32, where the change in position of Switch I region is highlighted with an arrow.

C) AlphaFold2-predicted RAB32:LRMDA assembly with annotation for the Switch I, Interswitch, Switch II and P-loop regions of RAB32 at the interface with LRMDA, and the location of residues, relevant for the interaction.

D) Comparison of active and inactive-RAB32 from panel (B) shows the localised change in flexibility of Switch I that occurs upon GTP binding. In inactive RAB32, residues that are important for LRMDA interaction (D61 and F62) are displaced.

E) Alignment of RAB32 and RAB38 showing the conservation of residues D-61, F-62 and L-64 (numbering for RAB32).

F) Alignment of AlphaFold-2 predicted models for the assembly of RAB32-LRMDA and RAB38-LRMDA.

G) Alignemnt of active RAB32 with active, GTP-bound, RAB38 (PDB: 6HDU) (McGrath et al., 2021) shows the similarity in conformation of predicted interfacial residues.

bioRxiv preprint doi: https://doi.org/10.1101/2025.02.04.636395; this version posted February 4, 2025. The copyright holder for this preprint (which was not certified by peer review) is the author/funder, who has granted bioRxiv a license to display the preprint in perpetuity. It is made available under aCC-BY 4.0 International license.

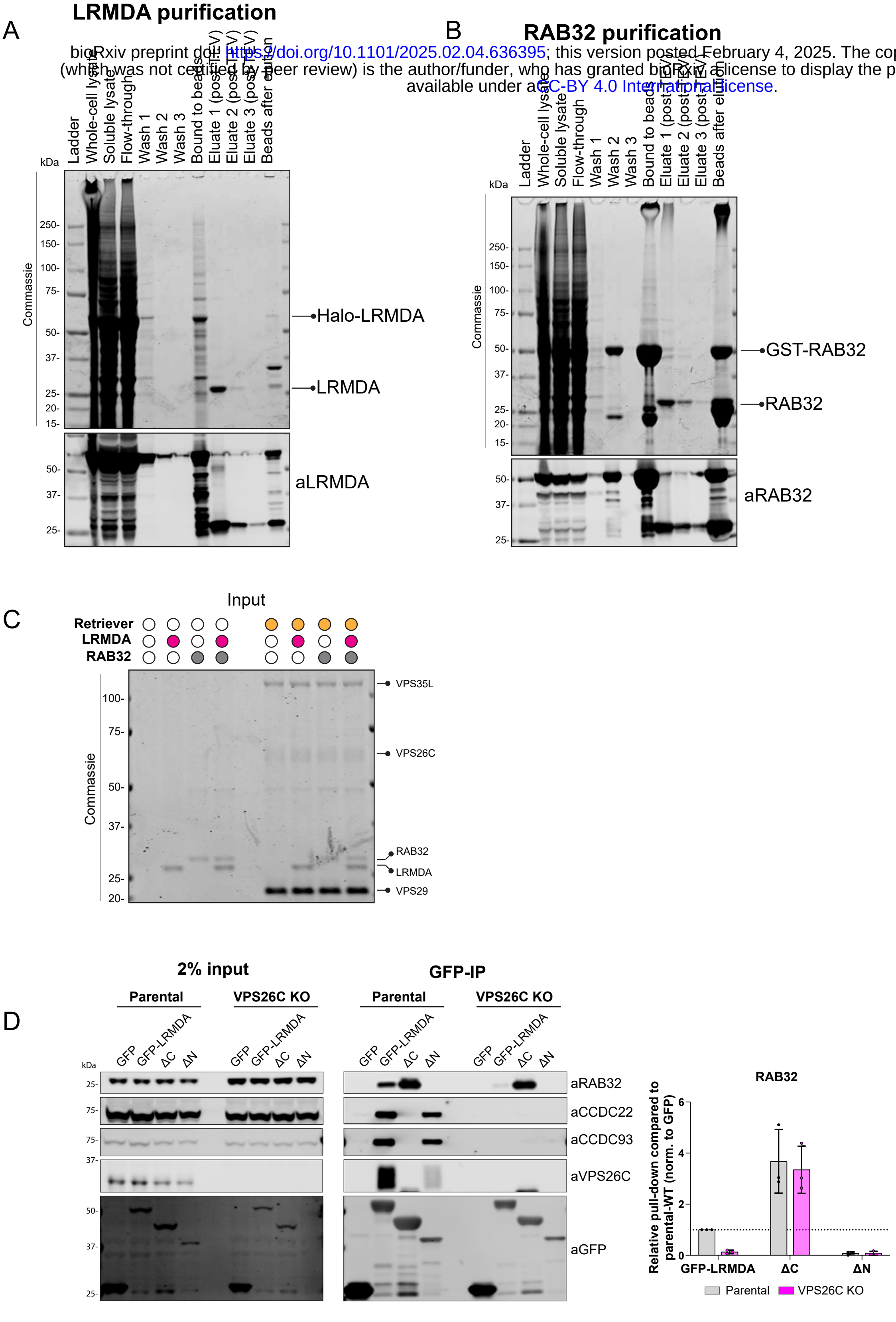

bioRxiv preprint doi: https://doi.org/10.1101/2025.02.04.636395; this version posted February 4, 2025. The copyright holder for this preprint (which was not certified by peer review) is the author/funder, who has granted bioRxiv a license to display the preprint in perpetuity. It is made available under aCC-BY 4.0 International license.

Supplementary Figure 2:

A) Halo-LRMDA was expressed in insect cells, and purified using HaloLink resin. TEV protease was used to remove the Halo Tag.

B) GST-RAB32 was expressed in insect cells, and purified using Glutathione Sepharose beads. TEV protease was used to remove the GST Tag.

C) Inputs for the experiment are shown in Figure 3E.

D) Parental or VPS26C KO HeLa cells were transfected with GFP-LRMDA or truncated forms of GFP-LRMDA, and the lysates were used in GFP-trap experiments to analyze the association with Retriever complex or RAB32. $n = 3$, 2-way ANOVA with Šídák's multiple comparisons test, data presented as mean values and error bars represent s.d.; n.s. denotes changes with $p > 0.05$.

Butkovic et al., 2025, Supplementary Figure 3

bioRxiv preprint doi: https://doi.org/10.1101/2025.02.04.636395; this version posted February 5, 2025. The copyright holder for this preprint (which was not certified by peer review) is the author/funder, who has granted bioRxiv a license to display the preprint in perpetuity. It is made available under aCC-BY 4.0 International license.

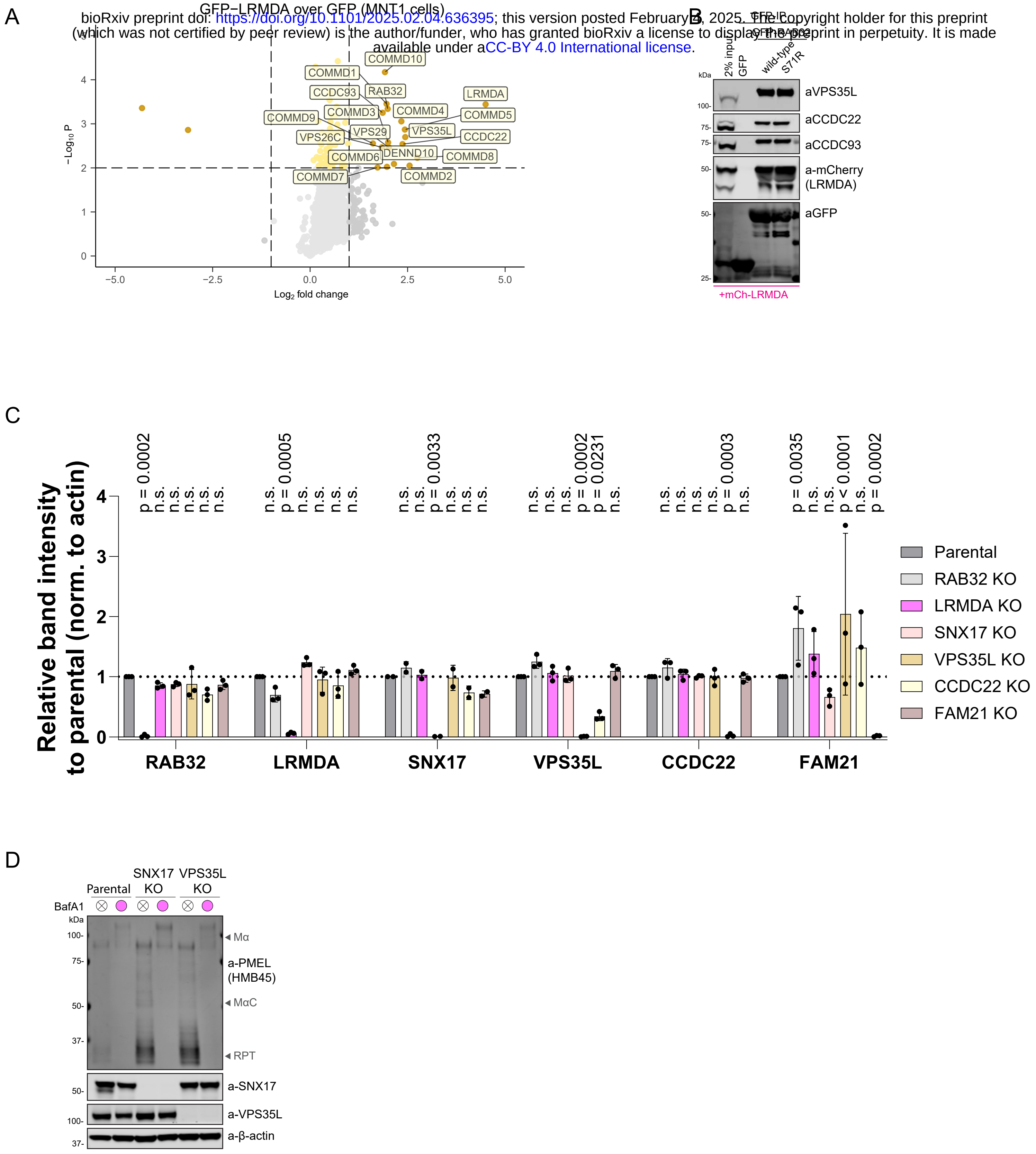

bioRxiv preprint doi: https://doi.org/10.1101/2025.02.04.636395; this version posted February 4, 2025. The copyright holder for this preprint (which was not certified by peer review) is the author/funder, who has granted bioRxiv a license to display the preprint in perpetuity. It is made available under aCC-BY 4.0 International license.

Supplementary Figure 3:

A) Volcano plot showing the enrichment of proteins in GFP-LRMDA pulldown in MNT1 cells, compared to the GFP sample. N=3, paired t-test, significantly enriched proteins with $p < 0.01$ and Log2 fold change > 1 are labelled.

B) HEK293T cells were transfected with GFP, GFP-RAB32 or Parkinson's disease-causative mutant GFP-RAB32 S71R. The lysates were used in GFP-trap experiments to analyze the association with Retriever complex or LRMDA.

C) Protein levels in whole-cell lysates (RIPA lysis buffer) for the experiment are shown in Figure 7B. $n = 3$, 2-way ANOVA with Dunnett's multiple comparison test, data presented as mean values and error bars represent s.d.; n.s. denotes changes with $p > 0.05$.

D) Protein levels in whole-cell lysates (RIPA lysis buffer) from parental or SNX17 KO or VPS35L KO cells treated with 100 nM BafA1 for 24h.

bioRxiv preprint doi: https://doi.org/10.1101/2025.02.04.636395; this version posted February 4, 2025. The copyright holder for this preprint (which was not certified by peer review) is the author/funder, who has granted bioRxiv a license to display the preprint in perpetuity. It is made available under aCC-BY 4.0 International license.

A

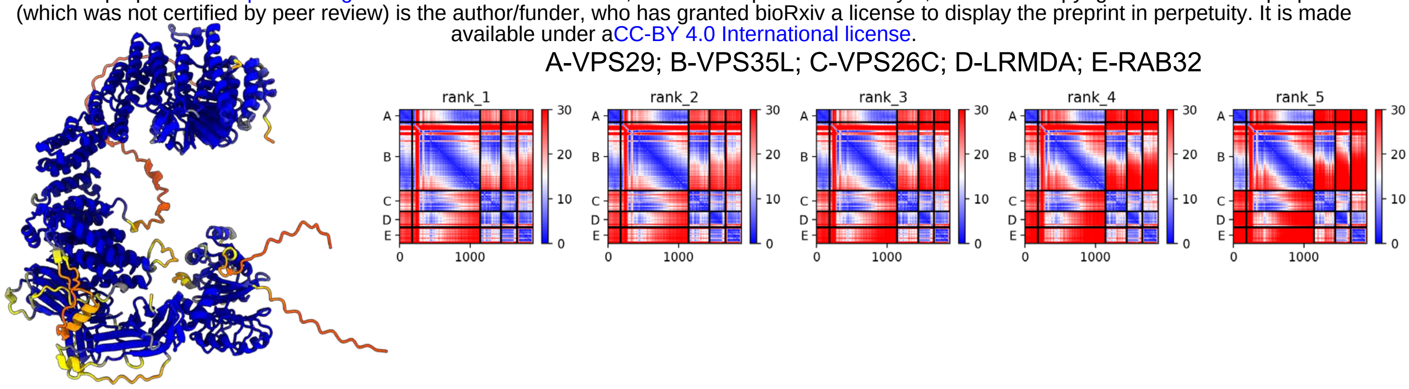

B

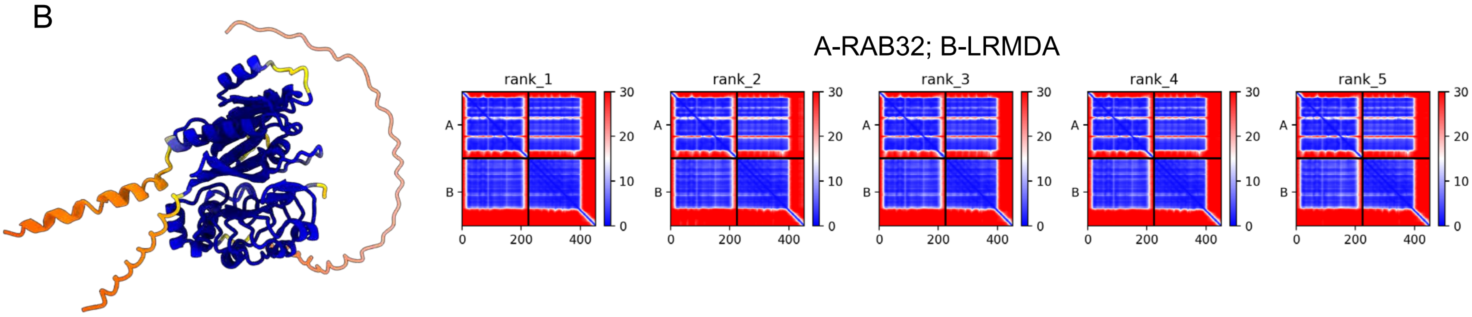

C

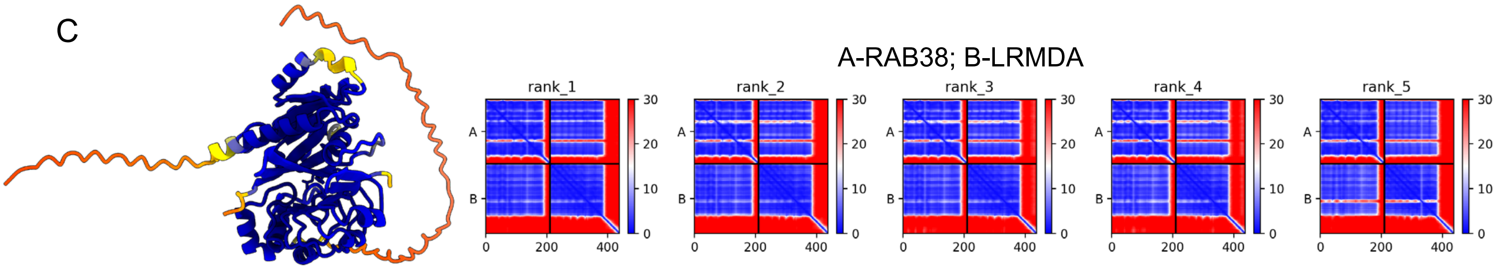

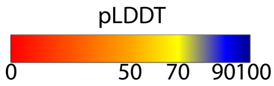

bioRxiv preprint doi: https://doi.org/10.1101/2025.02.04.636395; this version posted February 4, 2025. The copyright holder for this preprint (which was not certified by peer review) is the author/funder, who has granted bioRxiv a license to display the preprint in perpetuity. It is made available under aCC-BY 4.0 International license.

Supplementary Figure 4:

pLDDT scores and PAE plots for AlphaFold-2 predicted assemblies between LRMDA-RAB32-Retriever (A), LRMDA-RAB32 (B) and LRMDA-RAB38 (C).

bioRxiv preprint doi: https://doi.org/10.1101/2025.02.04.636395; this version posted February 4, 2025. The copyright holder for this preprint (which was not certified by peer review) is the author/funder, who has granted bioRxiv a license to display the preprint in perpetuity. It is made available under aCC-BY 4.0 International license.

Supplementary Data 1:

Uncropped western blot membranes and Commassie gels.

Supplementary Data 2:

Proteomic analysis datasets. *The data includes additional comparisons not discussed in the text using (i) a double GFP-LRMDA F145D+L226G mutant GFP-pulldown in RPE1 cells and (ii) GFP-LRMDA L226G mutant GFP-pulldown in MNT1 cells. These were used as negative controls for loss of binding to Commander complex components.*
